# The value of age IgG and IL6 in estimating time of viral clearance in asymptomatic or mild patients with COVID-19

**DOI:** 10.3389/fmicb.2023.1256759

**Published:** 2023-12-06

**Authors:** Xi Cao, Yong-Li Xie, Chun-lei Zhou, Hong Mu

**Affiliations:** ^1^Department of Clinical Laboratory, Tianjin First Central Hospital, Tianjin, China; ^2^Department of Clinical Laboratory, Tianjin Stomatological Hospital, School of Medicine, Nankai University, Tianjin, China; ^3^Tianjin Key Laboratory of Oral and Maxillofacial Function Reconstruction, Tianjin, China

**Keywords:** COVID-19, RT-PCR, nucleic acid clearance, IgG, IL6

## Abstract

**Background:**

The aim of this study was to investigate the relationship between Age, immunoglobin G (IgG), immunoglobin M (IgM), procalcitonin (PCT), and interleukin-6 (IL6), and the time to clear viral nucleic acids in asymptomatic and mild coronavirus disease 2019 (COVID-19) patients, as well as evaluated the predictive value of these biochemical indicators.

**Methods:**

We performed a retrospective analysis on 1,570 individuals who were admitted to Tianjin First Central Hospital and diagnosed with asymptomatic or mild cases. Laboratory data were collected, including age, gender, levels of IgG, IgM, PCT and IL6, as well as results of the severe acute respiratory syndrome coronavirus-2 (SARS-CoV-2) nucleic acid test. These data were statistically analyzed using SPSS software, version 24.0.

**Results:**

The results indicated that among mild patients, Age, IgG, and the time to clear viral nucleic acids were higher than asymptomatic patients (*p* < 0.05). And the time to clear viral nucleic acids was significantly correlated with Age, IgG, IgM, PCT, and IL6 (*p* < 0.05), IgG (*r* = −0.445, *p* < 0.001) showed moderate correlations. Using logistic regression analysis, we identified older age, high IL6 levels, and low IgG levels were risk factors for nucleic acid clearance exceeding 14 days (*p* < 0.05). When combining these three indicators to predict the probability of nucleic acid clearance exceeding 14 days in the 1,570 patients, the AUROC was found to be 0.727.

**Conclusion:**

Age, IgG, and IL6 could potentially serve as useful predictors for nucleic acid clearance exceeding 14 days in asymptomatic and mild COVID-19 patients.

## Introduction

The Coronavirus disease 2019 (COVID-19) is an infectious disease caused by a novel strain of viruses that belong to a family responsible for various illnesses ranging from the common cold to severe acute respiratory syndrome (SARS). The COVID-19 disease progression could lead to an overactive inflammatory response and cytokine storm, which can cause organ damage (Chen et al., 2020; Lopes-Pacheco et al., 2021). Studies have reported that high levels of viral RNA can be detected shortly after the onset of symptoms in COVID-19 patients, and these levels may persist for an extended period (Zou et al., 2020). There is growing concern that patients with prolonged viral infection may experience further organ or tissue injury as a result of the SARS-CoV-2 virus itself or ongoing inflammatory processes (Wang and Yang, 2022). Exploring the factors that influence virus clearance in patients is crucial for addressing chronic COVID-19 infection.

Recent studies have identified several risk factors for chronic viral infection, including older age (Crook et al., 2021; Notarte et al., 2022c), decreased humoral immune responses (Notarte et al., 2022a,b), and excessive production of inflammatory factors (Merad et al., 2022). As COVID-19 progresses, the levels of inflammatory factors in the body can significantly change, impacting the formation of immune responses during the early stages of the disease. Neutralizing immunoglobulin (IG) is capable of minimizing viral RNA replication and reducing the chances of disease progression by responding to the SARS-CoV-2 spike protein and/or its receptor-binding domain like the primary receptor angiotensin-converting enzyme 2 (ACE2) (Tegenge et al., 2021). Moreover, diminishing serum neutralizing activity or decreased antibody sensitivity has been linked to higher frequencies and longer time of COVID-19 infections (Gruell et al., 2022). Therefore the time it takes for SARS-CoV-2 nucleic acid to turn negative may be associated with cytokine storms and antibody levels induced by the viral infection.

Most published findings on COVID-19 have focused on critical patients, however there have been limited reports on patients with mild or asymptomatic cases, which make up the majority of infections. Asymptomatic or mild patients who experience long-term chronic viral infection are at a potential risk of developing severe symptoms. The objective of this article was to investigate relationship between important factors, including IgG, IgM, PCT, and IL6, and the time to clear viral nucleic acids. Additionally, we sought to assess the predictive value of these factors in determining the probability of nucleic acid clearance exceeding 14 days.

## Materials and methods

### Participants and data source

This study was conducted at Tianjin First Central Hospital in Tianjin, China, from September 2022 to June 2023. Inclusion criteria for this study were: (1) confirmed asymptomatic or mild COVID-19 cases, (2) nasopharyngeal swab test been performed more than twice within hospital stay, and the interval time > 24 h. Exclusion criteria for this study were: (1) patients with liver and/or kidney dysfunction, (2) patients with serious inflammatory diseases such as chronic pulmonary disease, digestive system disease, immunological diseases, et al., (3) patients with cerebral infarction or cardiovascular at the time of admission, or (4) patients with missing data. A total of 1,570 COVID-19 patients were included, with 743 being asymptomatic and 827 being mild cases. Data collection included recording symptoms at admission, laboratory results, and nucleic acid test outcomes from the COVID-19 rehabilitation ward at Tianjin First Central Hospital. The diagnosis of asymptomatic or mild cases was based on guidelines provided by the National Health Commission of China.

Serum IgG/IgM against the SARS-CoV-2 protein were evaluated by magnetic particle chemiluminescence (Biology and Science, China), PCT and IL6 were evaluated by quantitative electrochemiluminescence immunoassay (Ren mai, China). During the observation period, nasopharyngeal swabs were taken from all patients daily to minimize the occurrence of false-negative results. Each patient’s sample was tested using commercial kits for SARS-CoV-2 provided by two different manufacturers (Sheng Xiang/Bo Jie, China). The Ct value for each sample was calculated according to the manufacturer’s instructions, with a threshold Ct value of 40 set as positive according to the China Technical Guidelines for Laboratory Testing for COVID-19. The date of diagnosis was defined as the day when the first sample tested positive for SARS-CoV-2 by RT-PCR. Discharge criteria followed WHO guidance, requiring two consecutive negative PCR swabs taken more than 24 h apart, with no clinical symptoms.

According to the ninth edition of the China Novel Coronavirus Pneumonia Prevention and Control Protocol, COVID-19 patients were advised to be isolated for at least 14 days. So we set limit at 14 days, the patients were then divided into two groups: one group included patients whose SARS-CoV-2 nucleic acid turned negative within 14 days (*n* = 624), and the other group included patients whose SARS-CoV-2 nucleic acid turned negative after exceeding 14 days (*n* = 946). Demographic information, laboratory results, and chest CT scans were performed for all inpatients. These data were obtained from electronic medical records and reviewed by specialized physicians. This study was reviewed and approved by the Medical Ethics Committee of Tianjin First Central Hospital (Ethics Committee archiving No. 2022N052KY) and conformed to the principles outlined in the Declaration of Helsinki.

### Statistical analysis

Statistical analysis was performed with SPSS 24.0 for Windows software (SPSS, Chicago, IL). Normally distributed data were expressed as the mean ± standard deviation. We applied the t-test for between-group comparisons of variables that showed normal distribution of data with homogeneous variances. Non-normally distributed data are represented by the median (upper quartile and lower quartile) and we used the non-parametric Mann–Whitney U test for between-group comparisons of non-normally distributed data or data with heterogeneous variance. The categorical characteristics were described as counts and percentage (%). The relationship between biochemical indicators and prognosis were assessed by using Pearson or Spearman test. Risk factors were analyzed by univariate logistics regression, which described the direction of the relationship between predictor and the response variable, *p* value and odds ratio (OR) with its 95% confidence interval (CI). Significance corresponded to *p* < 0.05 and 95% CI for OR; 95% CI > 1 indicated risk of the event; whereas, 95% CI < 1 indicated protection. The performance of prognostic indicators were evaluated by the receiver operating characteristic (ROC) curves analysis. The value of AUROC closed to 1.0 indicates a high diagnostic accuracy. According to the principle that maximum of Youden index access to the best sensitivity and specificity. *p* < 0.05 was considered statistically significant, and *p* < 0.001 was considered highly statistically significant.

## Results

In total of 1,570 patients with COVID-19 (743 were asymptomatic patients and 827 were mild patients) were enrolled in the study. As shown in Table 1, the demographic and clinical characteristics of these patients were compared. The proportion of males were 52.22% in asymptomatic patients and 48.25% in mild patients. Significant difference was observed in age distribution between asymptomatic and mild patients, with mild patients being slightly older (37.52 ± 0.57 vs. 35.78 ± 0.65) (*p* < 0.05). There were no statistically significant differences in IgM, PCT, or IL6 levels between the two groups (*p* > 0.05). However, mild patients had higher levels of IgG and longer time to clear viral nucleic acids compared to asymptomatic patients, and these differences were statistically significant (*p* < 0.05).

**Table 1:** Laboratory characteristics of 1,570 asymptomatic or mild patients (mean ± SD).

Variables	Asymptomatic patients	Mild patients	*t* value	*p* value
Cases, *n*	743	827	/	/
Male, *n* (%)	388 (52.22%)	399 (48.25%)	/	/
Age (year)	35.78 ± 0.65	37.52 ± 0.57	−2.028	0.043
IgG (g/L)	18.69 ± 1.49	25.76 ± 1.71	−3.079	0.002
IgM (g/L)	0.49 ± 0.12	0.44 ± 0.08	0.360	0.719
PCT (ng/mL)	0.065 ± 0.0037	0.059 ± 0.0024	1.348	0.178
IL6 (U/ml)	12.37 ± 0.33	12.19 ± 0.33	0.386	0.700
Days of nucleic acid turn negative	15.86 ± 0.18	16.54 ± 0.19	2.641	0.008

We analyzed the correlation between biochemical indicators and days of nucleic acid turn negative. The correlation between time to clear viral nucleic acids and corresponding indicators in patients that are shown in Table 2. The results showed that correlation statistics of these indicators are statistically significant (*p* < 0.05). Age, IgM, PCT, and IL6 were weakly correlated with the time to clear viral nucleic acids (*r* = −0.144–0.151), however IgG was moderately correlated with the time to clear viral nucleic acids (*r* = −0.445). Based on the days for nucleic acid clearance, the patients were divided into two groups: those whose nucleic acid turned negative within 14 days (624 patients) and those whose nucleic acid turned negative after 14 days (946 patients). The data showed that patients whose nucleic acid turned negative within 14 days had lower age, lower IL6 levels, and higher IgG levels (*p* < 0.05). There were slight differences in PCT and IgM levels, but they were not statistically significant (*p* > 0.05) (Table 3).

**Table 2:** Correlation analysis of serum markers with the time of viral clearance.

Variables	Days of nucleic acid turned negative	
	*P* value	Correlation coefficient
Age (year)	<0.001	0.100
IgG (g/L)	<0.001	−0.445
IgM (g/L)	<0.001	−0.144
PCT (ng/mL)	0.034	0.054
IL6 (U/ml)	<0.001	0.151

**Table 3:** Characteristics differences of 1,570 COVID-19 patients between different groups.

Variables	Nucleic acid turned negative ≤14 days	Nucleic acid turned negative >14 days	*t* value	*p* value
Cases, *n*	624	946	/	/
Male, *n* (%)	329 (52.72%)	458 (48.41%)	/	/
Age (year)	34.24 ± 0.64	38.32 ± 0.57	−4.675	<0.001
IgG (g/L)	37.17 ± 2.50	12.68 ± 0.82	10.800	<0.001
IgM (g/L)	0.58 ± 0.15	0.38 ± 0.06	1.339	0.181
PCT (ng/mL)	0.058 ± 0.0031	0.064 ± 0.0029	−1.319	0.187
IL6 (U/ml)	11.11 ± 0.32	13.05 ± 0.33	−4.048	<0.001

We then used logistic regression analysis to evaluate the risk posed by these significant parameters in predicting the time to clear viral nucleic acids. The main factors influencing nucleic acid clearance exceeding 14 days were assessed (outcome of logistic regression: predictors and protectors of nucleic acid turned negative exceed 14 days). The results showed that IgG acted as a protective factor associated with nucleic acid clearance exceeding 14 days (OR = 0.786) (Table 4), while age, IgM, PCT, and IL6 were risk factors (OR = 1.516, 1.018, 1.307, and 1.617) (Table 4), and OR values calculation of Age, IgG, and IL6 were statistically significant (*p* < 0.05). A multivariate logistic regression model was built using these three variables, and it showed better predictive ability with an AUROC of 0.727 for predicting nucleic acid clearance exceeding 14 days.

**Table 4:** Influence factors associated with the time of viral clearance (univariate logistics regression).

Variables	Regression coefficient	Wald value	OR (95%CI)	*p* value	AUROC
Age	0.016	24.167	1.516 (1.510–1.523)	<0.001	0.595
IgG	−0.014	73.244	0.786 (0.783–0.789)	<0.001	0.626
IgM	0.018	0.880	1.018 (0.980–1.058)	0.348	0.480
PCT	0.268	0.158	1.307 (0.849–2.898)	0.691	0.530
IL6	0.017	6.407	1.617 (1.504–1.631)	0.011	0.610
Age + IgG + IL6	0.416	65.096	1.916 (1.614–2.215)	<0.001	0.727

## Discussion

SARS-CoV-2 is a novel SARS-related coronavirus with high transmissibility. During the early stage of illness, patients may underestimate their symptoms and delay seeking medical attention. Recent reports have shown that the median time to clear viral nucleic acids in COVID-19 patients is around 20 days, but it can be as long as 37 days (Zhou et al., 2020). Although many patients show improvement in clinical and radiographic manifestations over time, their viral load may still remain high (Magleby et al., 2021), prolonged viral clearance has been associated with unfavorable outcomes and organ injury (Al-Aly et al., 2022). Therefore, achieving rapid clearance of SARS-CoV-2 is desirable during the early stage of infection as it can help halt the dissemination of the virus in the body and limit tissue damage. In this analysis, we studied a large number of asymptomatic or mild COVID-19 patients and explored factors that influence the time to clear viral nucleic acids. We observed that the mild group tended to be older compared to the asymptomatic group. Additionally, they also took more days for their nucleic acid to turn negative. Through logistic regression analysis, we found that patients whose nucleic acid turned negative within 14 days had lower age and IL6 levels, as well as higher levels of IgG. When combining age, IgG, and IL6 as predictive factors, we were able to predict the likelihood of nucleic acid conversion exceeding 14 days roughly, with an AUROC value of 0.727.

Cytokines play a crucial role in regulating immune and inflammatory responses. Excessive production of certain cytokines, such as IL-6, has been identified as a major factor contributing to the inflammatory response observed in COVID-19 (Rabaan et al., 2021). It is produced by various cell types, including immune cells, endothelial cells, keratinocytes and tumor cells. IL6 serves as a proinflammatory cytokine capable of activating the intracellular Janus kinase (Jak)/signal transducer and activator of transcription (STAT) cascade and perpetuating inflammation through a STAT3-mediated positive feedback loop in non-immune cells. Clinical trials and reports had shown that IL-6 played an important role in the pathogenesis and severity of COVID-19 (Pelaia et al., 2021), inhibiting the IL6 receptor could effectively reduce mortality or the side effects of COVID-19 (Gupta et al., 2021). Our results demonstrated that high IL-6 levels was closely related with long time to clear viral nucleic acids, potentially due to the reduced efficiency of virus clearance caused by a cytokine storm. PCT is a marker commonly used to indicate bacterial infection and may be more effective than other clinical indicators such as C-reactive protein (CRP) and white blood cell count (WBC) (Becker et al., 2010). However, in our research, we found no significant difference in PCT levels between the mild patient group and the asymptomatic group. Additionally, PCT performed poorly in predicting virus clearance within the studied population (*p* > 0.05).

Neutralizing antibodies play a crucial role in providing protection against SARS-CoV-2 and are considered a key factor induced by COVID-19 vaccines. These antibodies have also been utilized in convalescent plasma therapy for antiviral treatment (Corti et al., 2021). Elevated levels of IgG antibodies can inhibit the inflammatory response in COVID-19 patients. They bind to the viral S-protein and interfere with its interaction with the ACE2 receptor, thus neutralizing the activity of SARS-CoV-2. Even low levels of IgG can be highly effective in the early stages of COVID-19 by opsonizing the virus and facilitating its clearance via Fc receptors (Marconato et al., 2022). Our finding aligns with this, revealing that patients with high serum IgG levels have a shorter viral clearance time. Higher IgG levels in patients during the early infection stage effectively suppressed viral replication and facilitated faster viral clearance. Interestingly, IgM did not demonstrate similar abilities. We supposed that IgG antibodies primarily play a significant role in viral clearance, particularly benefiting individuals in the early stages of infection. Antibody activities like IgG-driven neutralization and antibody-dependent cellular cytotoxicity had a greater impact on the rapid viral clearance than IgM.

In our study, we found that Age, IL6, and IgG levels have prognostic value in predicting prolonged time for nucleic acid conversion. This information can help us roughly determine which patients may take a shorter period of time to recover, providing valuable insights for clinical prevention and control strategies. However, this study has several limitations. Firstly, it is a retrospective study conducted in a single medical center. Secondly, viral mRNA was detected using a qualitative assay, lacking quantitative analysis for viral load calculation. Lastly, there are limited relevant studies available, and our research solely explored the correlation between several markers (IgG, IgM, PCT, and IL6) and the time of nucleic acid conversion. Future studies should comprehensively analyze additional laboratory test results.

## Data availability statement

The raw data supporting the conclusions of this article will be made available by the authors, without undue reservation.

## Ethics statement

The studies involving humans were approved by Medical Ethics Committee of Tianjin First Central Hospital. The studies were conducted in accordance with the local legislation and institutional requirements. The participants provided their written informed consent to participate in this study.

## Author contributions

XC: Conceptualization, Writing – original draft, Writing – review & editing, Data curation. Y-LX: Investigation, Software, Writing – original draft. C-lZ: Investigation, Software, Writing – original draft. HM: Project administration, Supervision, Writing – review & editing, Writing – original draft.
